# Delayed Rebound of Glycemia During Recovery Following Short-Duration High-Intensity Exercise: Are There Lactate and Glucose Metabolism Interactions?

**DOI:** 10.3389/fnut.2021.734152

**Published:** 2021-11-11

**Authors:** Laurent A. Messonnier, Benjamin Chatel, Chi-An W. Emhoff, Léo Blervaque, Samuel Oyono-Enguéllé

**Affiliations:** ^1^Laboratoire Interuniversitaire de Biologie de la Motricité, Université Savoie Mont Blanc, Chambéry, France; ^2^Cellmade Laboratories, Le Bourget-du-Lac, France; ^3^Department of Kinesiology, Saint Mary's College of California, Moraga, CA, United States; ^4^Laboratoire de Physiologie de la Faculté de Médecine, Université Yaoundé 1, Yaoundé, Cameroon

**Keywords:** lactate, glucose, recovery, gluconeogenesis, liver

## Abstract

Lactate constitutes the primary gluconeogenic precursor in healthy humans at rest and during low-intensity exercise. Data on the interactions between lactate and glucose metabolisms during recovery after short-duration high-intensity exercise are sparse. The aim of the present study was to describe blood glucose ([glucose]_b_) and lactate ([lactate]_b_) concentration curves during recovery following short-duration high-intensity exercise. Fifteen healthy Cameroonian subjects took part in the study and performed successively (i) an incremental exercise to exhaustion to determine maximal work rate (P_max_) and (ii) a 2-min 110% P_max_ exercise after which blood lactate and glucose concentrations were measured during the 80-min passive recovery. In response to the 2-min 110% P_max_ exercise, [glucose]_b_ remained stable (from 4.93 ± 1.13 to 4.65 ± 0.74 mmol^.^L^−1^, NS) while [lactate]_b_ increased (from 1.35 ± 0.36 to 7.87 ± 1.66 mmol^.^L^−1^, *p* < 0.0001). During recovery, blood lactate concentrations displayed the classic biphasic curve while blood glucose concentrations displayed a singular shape including a delayed and transitory rebound of glycemia. This rebound began at 27.7 ± 6.2 min and peaked at 6.78 ± 0.53 mmol^.^L^−1^ at 56.3 ± 9.7 min into recovery. The area under the curve (AUC) of [lactate]_b_ during the rebound of glycemia was positively correlated with the peak value of glycemia and the AUC of [glucose]_b_ during the rebound. In conclusion, the delayed rebound of glycemia observed in the present study was associated with lactate availability during this period.

## Introduction

For almost a century, lactate has been known as a major gluconeogenic precursor. Indeed, numerous studies observed a splanchnic uptake of lactate during exercise and subsequent recovery (1–4). The Cori (or lactate) cycle, originally described in 1929, involves the conversion of glucose (and/or glycogen) to lactate in the skeletal muscle and the conversion back to glucose (and/or glycogen) from lactate in the liver (5). These glucose-lactate metabolism interactions have been observed in various clinical contexts. For instance, deficient gluconeogenesis may induce lactate accumulation (6) and in patients with acute myocardial infarction, glycemia was directly related to plasma lactate (7). In healthy subjects, although it has been demonstrated that the main fate of lactate is oxidation, lactate serves as the primary gluconeogenic precursor (8–12). This is true at rest, during moderate-intensity exercise, and also during recovery (8–12). On the one hand, liver (and to a lesser extent kidney) gluconeogenesis from lactate is driven by lactate delivery (dependent on blood lactate concentrations and local splanchnic blood flow), fractional extraction, hormonal milieu (including catecholamines, glucagon, and cortisol), sensitivity to these hormones, acidosis, and gluconeogenic pathway enzyme activity. On the other hand, gluconeogenesis may also be driven by the peripheral glucose demand from the exercising or previously active muscles, as a result of exercise duration and intensity, and consequently the metabolic activity of different fiber types (4, 13–17). In untrained as well as in trained subjects, exogenous lactate infusion (via a lactate clamp procedure) during endurance exercise augments gluconeogenesis, significantly increasing the gluconeogenic proportion of glucose rate of appearance during exercise (18, 19). Furthermore, endurance training improves gluconeogenesis from lactate by two-fold at rest and by three-fold during moderate-intensity exercise (8). Altogether, while glucose and lactate metabolism interactions, as part of the lactate shuttle concept (20), are well-documented in the literature at rest, during moderate-intensity exercise, and its subsequent recovery (21), data during recovery following short-duration high-intensity exercise are, by contrast, sparse.

During high-intensity exercise, glycogenolysis and glycolysis are highly activated, leading to major lactate production (22–24), and consequently muscle and blood lactate accumulations (25, 26). During passive recovery following this type of exercise, lactate concentrations may remain elevated (higher than resting levels) for approximately 60 min, depending on duration and work rate of the previously performed exercise, as well as on the training state of the subjects (27, 28). Circulating lactate feeds the liver to become a precursor for gluconeogenesis, thus begging the question of whether glucose and lactate metabolism interactions occur during passive recovery following high-intensity exercise.

The aim of the present preliminary study was to investigate the blood glucose curve during recovery following high-intensity exercise. Because it is known that (i) this type of exercise leads to a significant endogenous lactate production and accumulation, and (ii) lactate availability drives hepatic gluconeogenesis, interactions between lactate and glucose metabolism are suspected.

## Methods

### Subjects

Fifteen male Cameroonian volunteers (six active subjects and nine college football players) participated in the study. Their [mean ± standard deviation (SD)] age, height, and weight were 23 ± 2 years old, 173 ± 4 cm, and 67 ± 5 kg, respectively. Prior to giving their written consent, subjects were informed of the aim and potential risks or discomforts associated with the experiments. The experimental protocol was approved by the local ethics committee of the University of Yaoundé 1 (no. 10–12-2005) and was performed in accordance with the guidelines set by the Declaration of Helsinki for human studies.

### Experimental Design

The experiments took place at the General Hospital of Yaoundé (Cameroon). The protocol included two visits consisting of an incremental exercise to exhaustion (visit 1) and a short-duration high-intensity exercise (visit 2). For each visit, the subjects arrived either at 8:00 a.m. or 12:00 p.m. and had a light standardized breakfast or lunch followed at least by 90 or 150 min of rest (respectively) on site before exercising. The standardized breakfast or lunch was given to the subjects to ensure they were fed and not hypoglycemic at the moment of the exercise. The delay before exercise was to avoid the normal glycemic response immediately following food intake (29). An euglycemic pre-exercise glucose measurement was confirmed before exercise testing on the second visit when glucose recovery curves were recorded.

#### Visit 1: Incremental Exercise to Exhaustion

Subjects performed an incremental exercise test to volitional exhaustion using a leg-cycle ergometer (Ketler, Ense-Parsit, Germany) as previously described (30). The exercise started at 70 W for 3 min, after which the work rate increased by 35 W every 3 min thereafter. The exercise stopped when the subjects were no longer able to sustain the work rate and the requested cycling cadence of 70 rpm. Heart rate (HR; beats^.^min^−1^) was measured continuously using a chest monitor (Polar Electro, Kempele, Finland). This exercise session was used for determination of maximal HR (HR_max_; beats^.^min^−1^) and the work rate associated with HR_max_ (P_max_; W), which was estimated by linear interpolation from the HR vs. work rate curve.

#### Visit 2: Short-Duration High-Intensity Exercise

Upon subjects' arrival to the lab, after the standardized meal and resting period, a blood micropuncture (20 μL) was collected from the earlobe. Then, the volunteers performed a 10-min warm-up at a heart rate of 130 beats^.^min^−1^ (~50% of P_max_). After a 5-min rest, the subjects cycled for 2 min at 110% of P_max_. Capillary blood sampling (20 μL) were collected from the earlobe at exercise completion, and thereafter at 0.5, 1, 1.5, 2, 2.5, 3, 3.5, 4, 4.5, 5, 6, 8, 10, 12, 15, 20, 25, 30, 40, 50, 60, 70, and 80 min of passive recovery. Blood samples were used to determine glucose ([glucose]_b_; mmol^.^L^−1^) and lactate ([lactate]_b_; mmol^.^L^−1^) concentrations by enzymatic method using a YSI 2300 STAT PLUS analyser (YSI Incorporated, Yellow Springs, OH, USA). Time courses of lactate and glucose concentrations during recovery were analyzed.

### Data and Mathematical Analyses

**Figure 1:**
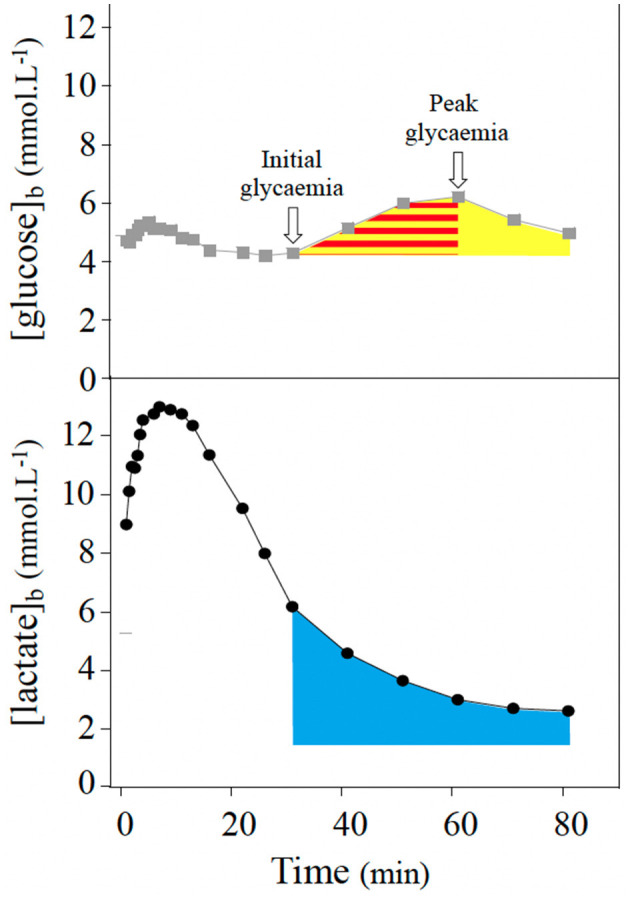
Analysis of blood glucose ([glucose]_b_, gray squares) and lactate ([lactate]_b_, black dots) concentration curves during recovery. Initial and peak values of glycemia during the delayed rebound of glycemia were considered. Area under the curve (AUC, calculated based on the trapezoidal rule) of the glycemic rebound (yellow area), of the increase part of the glycemic rebound (red area) and of lactatemia curve during the rebound period (blue area) were also considered.

Because the [glucose]_b_ curve during the recovery displayed a particular shape (Figure 1), initial and peak glycemia values during the delayed rebound were considered. Areas under the curve (AUC) integrating with the trapezoidal rule were calculated for each subject. The AUC of the delayed glycemic rebound (Figure 1, yellow area), the AUC between the initial and peak glycemia values of the rebound (Figure 1, red area), and the AUC for lactatemia during the delayed glycemic rebound (calculated above the resting [lactate]_b_, Figure 1, blue area) were considered.

Blood lactate recovery curves were fitted by Equation (1) using an iterative non-linear regression technique (Kaleidagraph 3.6, Synergy Software, PA, USA).


(1) $$\begin{array}{r} \left[lactate\right]\left(t\right)={\left[lactate\right]}_{\left(0\right)}+A_{1}\left(1-e^{{-\gamma }_{1}.t}\right)+A_{2}\left(1-e^{{-\gamma }_{2}.t}\right) \end{array}$$

Where [lactate]_(0)_ is [lactate]_b_ at exercise completion (onset of recovery, mmol^.^L^−1^), *t* is time (min), A_1_ and A_2_ are the amplitudes of the experimental terms (mmol^.^L^−1^) describing lactate appearance and disappearance in blood, respectively, and γ_1_ and γ_2_ the velocity constants (min^−1^) describing lactate exchange and removal abilities, respectively.

### Statistical Analysis

Descriptive statistics are means ± standard deviations. Correlations between different variables were studied by means of linear regression techniques (StatView 5.0, SAS, Cary, NC). The level of statistical significance was set at α ≤ 0.05.

## Results

### Incremental Exercise

During incremental exercise to exhaustion, HR_max_ and P_max_ were 184 ± 12 beats^.^min^−1^ and 203 ± 25 W (3.1 ± 0.4 W^.^kg^−1^), respectively.

### Two-Min 110% P_Max_ Exercise

**Figure 2:**
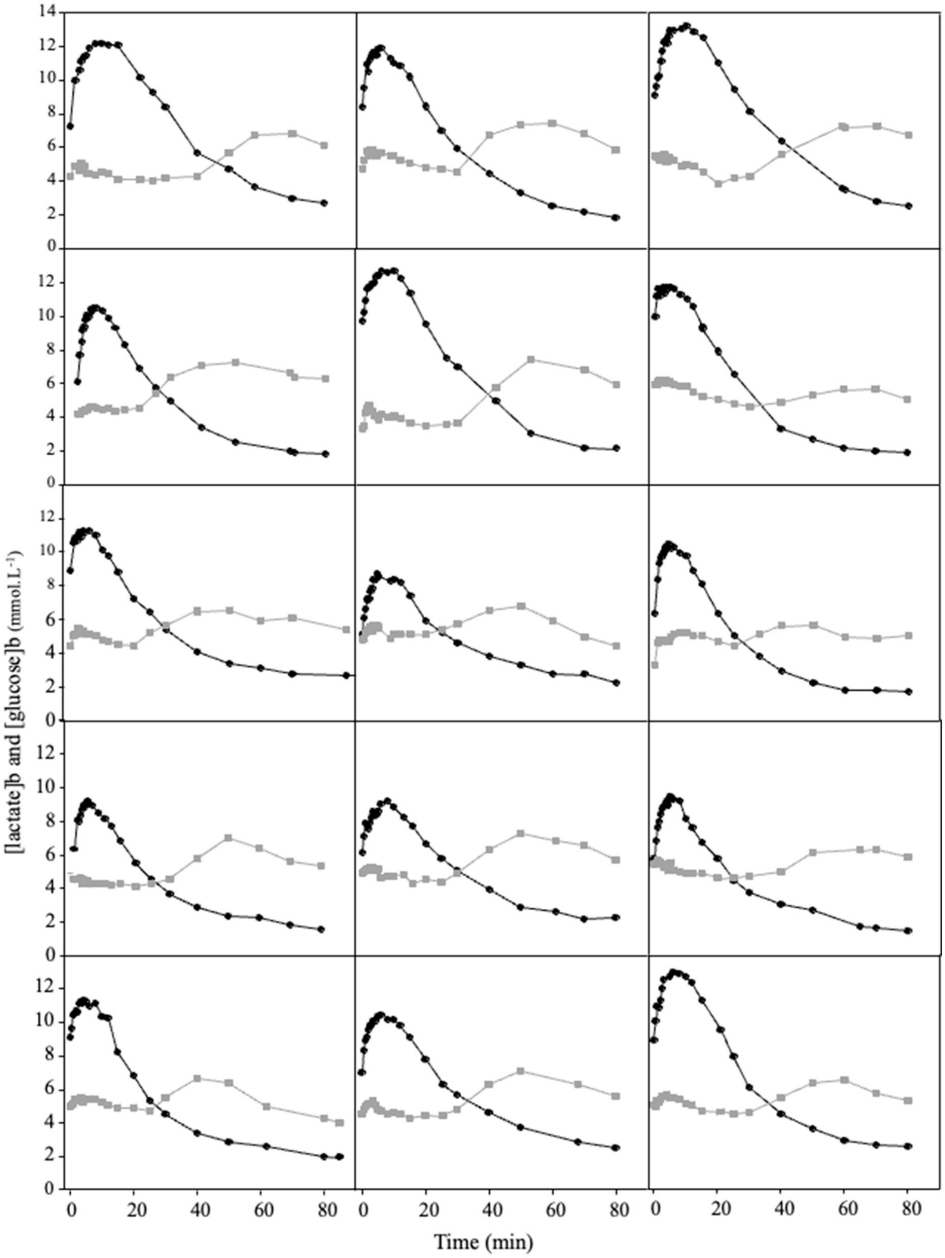
Blood glucose ([glucose]_b_, gray squares) and lactate ([lactate]_b_, black dots) concentration curves during recovery following 2-min 110% P_max_ (work rate associated with maximal heart rate) exercise obtained in the 15 subjects of the present study.

Figure 2 depicts blood glucose and lactate recovery curves following the 2-min 110% P_max_ exercises. At rest and at exercise completion, [glucose]_b_ was 4.93 ± 1.13 and 4.65 ± 0.74 mmol^.^L^−1^, respectively (NS). The blood glucose curves displayed a singular shape, in which after a brief and transitory burst followed by a return to near resting blood glucose concentration, a delayed rebound was observed. This rebound started 27.7 ± 6.2 min into recovery, at which time glycemia (termed initial glycemia) was 4.48 ± 0.34 mmol^.^L^−1^ (Figure 2). During the rebound, glycemia peaked at 6.78 ± 0.53 mmol^.^L^−1^ at 56.3 ± 9.7 min into recovery. The Δ glycemia (peak glycemia – initial glycemia at the onset of the rebound) was 2.30 ± 0.76. mmol^.^L^−1^. Area under the curve of the rebound as well as AUC between the initial and peak glycemia values of the rebound were 86.67 ± 36.91 and 41.35 ± 17.37 mmol^.^L^−1.^min, respectively.

At rest, [lactate]_b_ was 1.35 ± 0.36 mmol^.^L^−1^. At exercise completion, [lactate]_b_ ([lactate]_(0)_) reached 7.87 ± 1.66 mmol^.^L^−1^. Following exercise, the blood lactate recovery curves displayed the classic biphasic shape observed after short-duration high-intensity exercise (Figure 2). After a rapid increase, blood lactate concentrations peaked 5.73 ± 2.23 min into recovery at 11.07 ± 1.49 mmol^.^L^−1^. Thereafter, [lactate]_b_ decreased slowly to return to near resting levels after 80 min of recovery. Calculated above resting [lactate]_b_, AUC of the [lactate]_b_ curve during the delayed rebound of glycemia was 126.7 ± 54.8 mmol^.^L^−1.^min. Mean A_1_, γ_1_, A_2_, and γ_2_ values obtained from the fits were 11.39 ± 5.01 mmol^.^L^−1^, 0.217 ± 0.083 min^−1^, −17.56 ± 5.8 mmol^.^L^−1^, and 0.0528 ± 0.0128 min^−1^, respectively.

Blood lactate and glucose curves crossed at 5.1 ± 0.4 mmol^.^L^−1^ at 32.0 ± 6.8 min into recovery.

**Figure 3:**
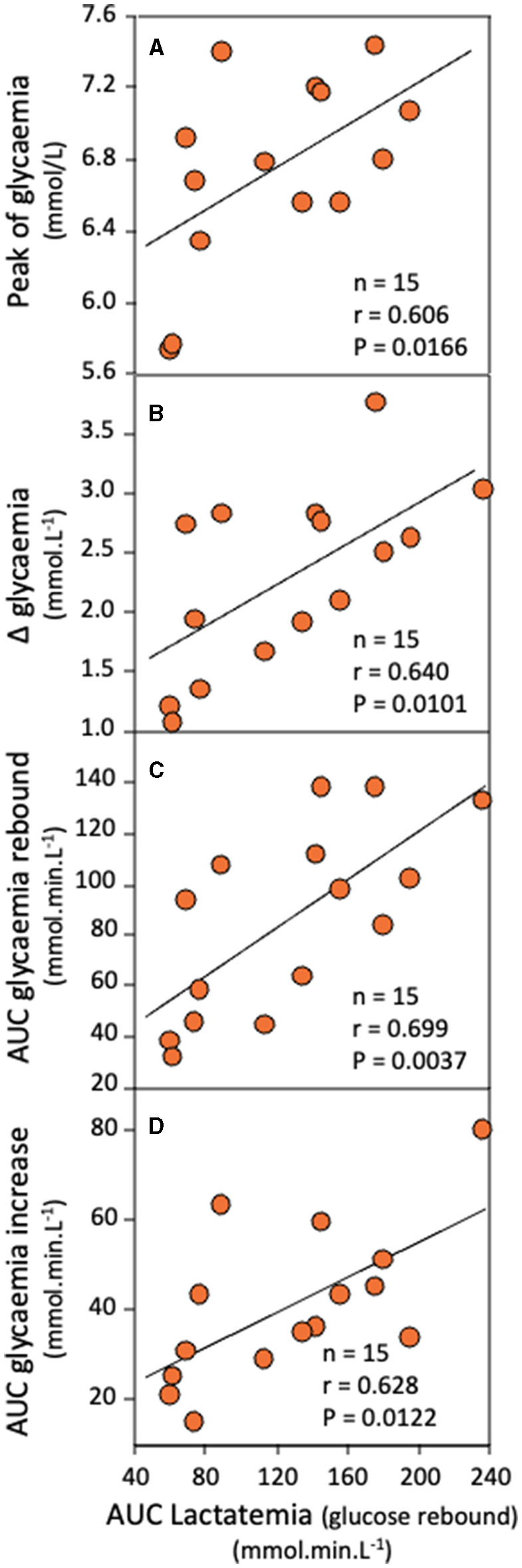
Correlations between AUC of [lactate]_b_ during the rebound of glycemia and the peak value of glycemia during the rebound **(A)**, Δ glycemia during the rebound **(B)**, the AUC of glycemia during the rebound **(C)**, and the AUC between the initial and peak glycemia values of the rebound **(D)**.

**Figure 4:**
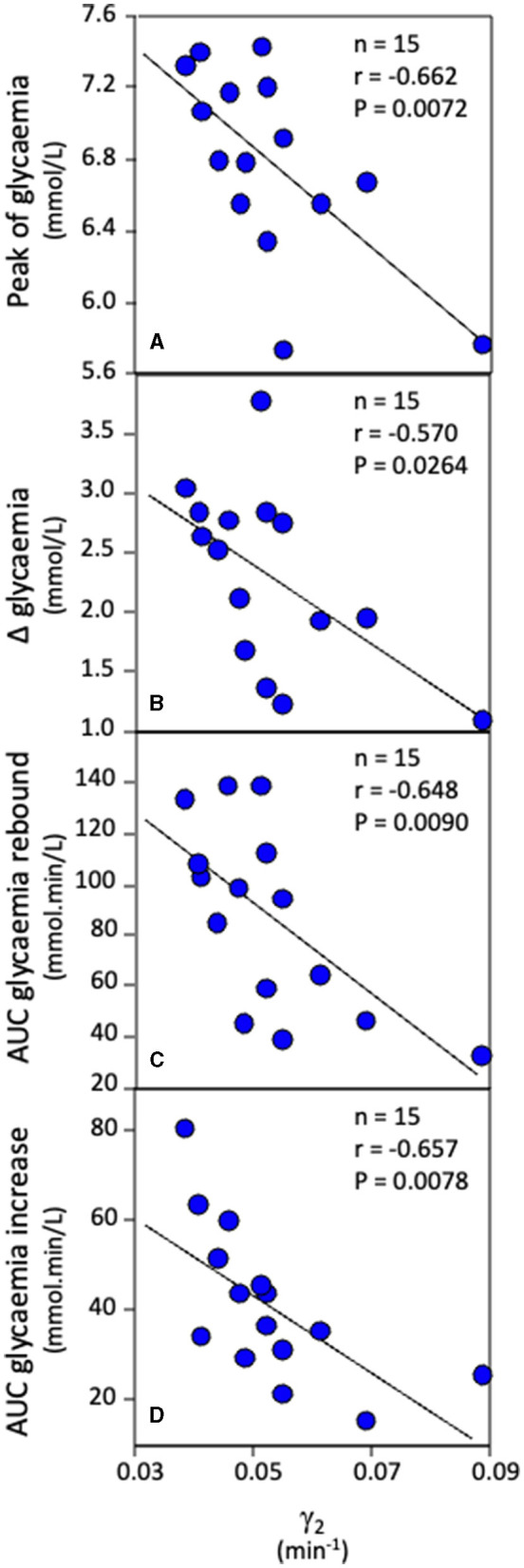
Correlations between γ_2_ and the peak value of glycemia during the rebound **(A)**, Δ glycemia during the rebound **(B)**, the AUC of glycemia during the rebound **(C)**, and the AUC between the initial and peak glycemia values of the rebound **(D)**.

Correlational analyses revealed that AUC of the [lactate]_b_ curve during the delayed rebound of glycemia (Figure 1) was positively correlated and γ_2_ was inversely correlated with the peak value of glycemia during the rebound (Figures 3A, 4A, respectively), the Δ glycemia during the rebound (Figures 3B, 4B), the AUC during the rebound (Figures 3C, 4C), and the AUC between the initial and peak glycemia values of the rebound (Figures 3D, 4D).

## Discussion

The aim of the present study was to describe the blood glucose recovery curve in response to short-duration high-intensity exercise and to investigate possible interactions with that of lactate. If the glycemic burst in the initial phase of recovery has already been reported, the main finding of the present study is the presence of a transitory glycemic rebound starting 20–45 min into recovery. Several parameters of this delayed glycemic rebound were correlated with area under the curve (AUC) of lactatemia at the time of the rebound.

### Initial Burst

The initial burst of glycemia in the early phase of recovery (peak of 5.36 ± 0.49 mmol^.^L^−1^ at 3.3 ± 1.7 min, and end at 8–20 min into recovery; Figure 2) is consistent with previous studies in healthy humans (4, 12, 31–33). The shorter burst observed in the present study might be attributed to the higher intensity and especially shorter duration of exercise (2 min), which very likely induced less metabolic challenges (e.g., hormonal milieu change, muscle glycogen depletion, muscle glucose need) than the much longer exercises performed in the former studies (peak of 4.52–7.40 mmol^.^L^−1^ at 4–10 min, and end at 20–60 min into recovery) (4, 12, 31–33). Wahren et al. (4) attributed these high levels of glycemia after exercise completion to the fact that if splanchnic glucose output decreased at the conclusion of exercise, the fall in peripheral glucose consumption after cessation of exercise was even faster, resulting in a transitory rise in glycemia.

### Delayed Rebound of Glycemia: Interaction With Lactate Metabolism?

To our knowledge, this is the first time a delayed rebound of glycemia is observed during recovery following short-duration high-intensity exercise. Actually, this rebound corresponds to a transitory hyperglycemia, reaching 6.78 ± 0.53 mmol^.^L^−1^ at 56.3 ± 9.7 min into recovery. Although Chiolero et al. (34) had observed a similar delayed and transitory rebound of glycemia approximately 30 min after a bolus infusion of exogenous sodium lactate leading to approximately 10.4 mmol^.^L^−1^ of [lactate]_b_ in partially hepatectomized patients, the divergences between the studies do not allow to infer from this previous study to explain our results.

Our observations beg the question for the mechanisms inducing such a delayed rebound. The rebound of glycemia indicates that glucose production becomes transiently higher than glucose uptake. In other words, the rebound may be due either to a decrease in glucose uptake, or to an increase in glucose production, or to a combination of these two possibilities. Although the potential underlying mechanisms of the rebound have not been studied here, the literature may provide some clues.

Concerning a possible decrease in glucose uptake, Åstrand et al. (12) observed that after a short maximal exercise, the arterial-femoral venous difference of glucose across the previously active legs is elevated in the first 15–20 min of recovery and then returned to resting levels. Taking into consideration that after short maximal exercise, muscle blood flow remains also elevated until approximatively 20 min into recovery (25), these results indicate that the uptake of glucose by the previously active muscle decreased drastically approximately 20 min after exercise completion (returning to very similar values as rest). Therefore, it cannot be totally excluded that a decrease in muscle glucose uptake would account at least partly for the rebound observed in the present study. However, it is unlikely that a decrease in glucose uptake by the previously active muscles accounts *per se* for the entire rebound observed. Therefore, an increase in glucose appearance can also be hypothesized.

Concerning a possible increase in glucose production, several hypotheses can be evoked. Because glycemia is normally subjected to daily variations due to energy intake, a first hypothesis would be that the rebound was related to a previous meal, as the concentrations reported here during the rebound mirror those obtained in response to a regular meal (29). Although this possibility cannot be totally excluded, it remains highly unlikely. Ando et al. (29) demonstrated that blood glucose peaked 30–60 min after the meal, while in the present study, exercise tests occurred at least 90–150 min after the breakfast or lunch, respectively. A second hypothesis would be that an increase of glucose output is associated with interactions between glucose and lactate metabolism. In the present study, positive correlations were observed between the AUC of lactatemia during the glycemic rebound and different indices of the glycemic rebound such as the peak value of glycemia during the rebound (Figure 3A), the difference between glycemia at the peak and at the onset of the rebound (Δ glycemia, Figure 3B), the AUC of the rebound (Figure 3C) and the AUC between the initial and peak glycemia values of the rebound (Figure 3D). These correlations support the idea that lactate availability may account, at least partly, for the rebound of glycemia observed here and that lactate and glucose metabolism interactions may exist during this period. This second possibility is supported by numerous studies in the literature which underline the role of lactate as a major precursor for gluconeogenesis by the liver and, to a lesser extent, by the kidneys (21, 34). In a previous report, Chiolero et al. (34) infused sodium lactate and modeled lactate decay post infusion by two exponential terms in partially hepatectomized patients. The greater the mass of liver excision in hepatectomized patients, the slower the half-life of the exponential term attributed to hepatic gluconeogenesis. This latter correlation further underlines the link between liver metabolism and lactate disappearance following a hyperlactatemia. If lactate is available, its delivery to the liver depends also on the local blood flow. In that regard, it is interesting to observe that estimated splanchnic blood flow, which was drastically decreased in response to short-duration high-intensity exercise, returned gradually to the resting value, reached 45 min into recovery (12). This gives support to the hypothesis of a possible elevated uptake of lactate by the liver (and/or the kidney) during the late period of the recovery. Besides, the high-intensity exercise performed in the present study is known to induce a significant adrenaline and noradrenaline secretion (35, 36), and adrenaline is known to activate gluconeogenesis (13, 37). The question is to know whether this catecholaminergic response may account for a delayed glucose output and rebound of glycemia. Interestingly, Ahlborg and Felig (2) observed that if catecholamine levels fell after exercise cessation, their concentrations were still 2.0–2.5 times the basal values 40 min after exercise (*P* < 0.01). Along with catecholamines, “elevated glucagon which develops during exhaustive exercise, persists” beyond 30 min of recovery (20, 38, 39). Taken together, these results suggest that gluconeogenesis might be still activated for a long time into recovery.

In the present study, inverse correlations have been observed between γ_2_ (the velocity constant denoting the lactate removal ability during the recovery) and the peak value of glycemia during the rebound, the Δ glycemia during the rebound, the AUC of glycemia during the rebound, and the AUC between the initial and peak glycemia values of the rebound (Figure 4). Because previous studies have related γ_2_ to the previously active muscles' oxidative capacity (citrate synthase activity and mitochondrial respiration) and content of MCT1 (involved in lactate uptake by the muscle) (40, 41), γ_2_ is believed to be mainly attributed to oxidation of lactate during the recovery. Therefore, these correlations suggest that the faster the disappearance of lactate during the recovery by oxidation, the smaller the rebound of glycemia. Taken together, all the correlations found in the present study (Figures 3, 4) provide support for the hypothesis that the rebound of glycemia is related to glucose and lactate metabolism interactions. The putative underlying mechanisms is that lactate would be used for gluconeogenesis, inducing a delayed and transient glucose output.

## Limitations and Perspectives

The present study has been performed in Cameroonian natives. Therefore, one cannot exclude that the present results were at least partially determined by ethnic, genetic, or epigenetic modulations (42). In accordance with such a hypothesis, Osei et al. (43) demonstrated that during fasting or after oral glucose ingestion, insulin levels were higher in West African (Ghanaian) natives than in immigrant counterparts, healthy African-Americans or white Americans. Therefore, it is uncertain whether such particular blood glucose recovery curves would be observed in other situations and populations. We also acknowledge that the metabolic status at the time of the exercise can be different even if a standardized breakfast or lunch is given to the subject before exercise.

If the literature reports that the liver is the major lactate recycler to glucose, one cannot exclude or underestimate the possible role of the kidneys. Of course, the present study cannot discern the respective importance of the two organs in the observed glycemic rebound.

In the discussion, several explanations have been put forward to explain the delayed rebound of glycemia (muscle and splanchnic blood flow, catecholamines, glucagon) and several others could have been evoked (e.g., cortisol, acidosis, muscle fiber types, and their metabolism). However, these explanations found in the literature have been obtained in contexts (after exercises) relatively different than the one of the present study. Therefore, caution should be taken and further studies are warranted to determine the exact underlying metabolic and hemodynamic mechanisms of the observed delayed and transient rebound of glycemia.

## Conclusion

The present study reports preliminary results showing a delayed rebound of glycemia during passive recovery following short-duration high-intensity exercise. The results of the present study (correlations) and literature lead us to suspect interactions between lactate and glucose metabolisms and especially a delayed and transient increase in glucose output. However, further mechanistic studies are necessary to test this hypothesis.

## Data Availability Statement

The raw data supporting the conclusions of this article will be made available by the authors, without undue reservation.

## Ethics Statement

The studies involving human participants were reviewed and approved by Local Ethics Committee, Medical School, University of Yaoundé 1 (no. 10-12-2005). The patients/participants provided their written informed consent to participate in this study.

## Author Contributions

LM and SO-E designed and conducted the study. LM and BC analyzed data. LM wrote the first draft of the article. All authors (except SO-E who died since the experiments) critically reviewed the draft and approved the final version for publication.

## Conflict of Interest

BC was employed by Cellmade Laboratories. LM receives consulting fees from Abbott Diabetes Care. The remaining authors declare that the research was conducted in the absence of any commercial or financial relationships that could be construed as a potential conflict of interest.

## Publisher's Note

All claims expressed in this article are solely those of the authors and do not necessarily represent those of their affiliated organizations, or those of the publisher, the editors and the reviewers. Any product that may be evaluated in this article, or claim that may be made by its manufacturer, is not guaranteed or endorsed by the publisher.
